# When Breast Cancer Meets the Uterus: A Quantitative Review of 105 Cases Spanning Four Decades

**DOI:** 10.3390/medicina62061205

**Published:** 2026-06-22

**Authors:** Tiberiu Augustin Georgescu, Antonia Carmen Georgescu, Maria Victoria Olinca

**Affiliations:** 1 Discipline of Pathology, “Carol Davila” University of Medicine and Pharmacy, 020021 Bucharest, Romania; tiberiu.georgescu@umfcd.ro (T.A.G.); maria.olinca@umfcd.ro (M.V.O.); 2 Department of Pathology, National Institute for Mother and Child Health “Alessandrescu-Rusescu”, 020395 Bucharest, Romania; 3 Department of Pathology, “Carol Davila” Nephrology Hospital, 010731 Bucharest, Romania

**Keywords:** breast carcinoma, uterine neoplasms, invasive lobular carcinoma, endometrial metastasis, endometrial polyp, tamoxifen, immunohistochemistry, GATA3, TRPS1, E-cadherin

## Abstract

*Background and Objectives:* Uterine metastasis from breast carcinoma is rare but poses substantial diagnostic and therapeutic challenges. Invasive lobular carcinoma (ILC) demonstrates a documented predilection for unusual metastatic patterns including the female genital tract, while tamoxifen-associated endometrial pathology may complicate diagnosis in breast cancer survivors. *Materials and Methods:* We performed a structured PubMed/MEDLINE and Google Scholar search (1980–2025) for cases with histologically confirmed breast primary and uterine involvement; a pooled analysis of demographic, histological, molecular, and clinical variables was performed. *Results:* 105 individual cases were identified. ILC accounted for 58.0% of histologically classified cases despite representing only 10–15% of breast cancers. Endometrial involvement was present in 68.6%, myometrial in 25.7%, and cervical in 26.7%. Tamoxifen exposure was strongly associated with polyp-substrate metastasis (29.3% vs. 4.7%; Fisher’s exact *p* = 0.0009; OR 8.41, 95% CI 2.20–32.14). Abnormal uterine bleeding was the dominant presentation (68.1%); 19.8% were asymptomatic. Median latency was 36 months (range from 24 months before to 360 months after the breast cancer diagnosis). *Conclusions:* Uterine metastasis from breast carcinoma is dominated by invasive lobular histology and frequently involves tamoxifen-associated polyps. A combined immunohistochemical panel (GATA3, TRPS1, E-cadherin, hormone receptors, PAX8) is essential for distinguishing metastatic disease from primary uterine pathology. Endometrial sampling should be considered with a low threshold in breast cancer survivors with abnormal uterine bleeding, and breast imaging is warranted when discohesive cells are encountered without a known breast primary. These proportions describe the published case literature rather than population-based prevalence; because the evidence is limited to case reports and small series, they should not be read as the true frequency of uterine involvement among women with breast cancer.

## 1. Introduction

Breast carcinoma is the most common malignancy in women globally and the leading cause of cancer-related death among women in many parts of the world. The natural history of metastatic spread is most often dominated by haematogenous dissemination to the bones, lungs, liver, and brain, with these four sites accounting for the great majority of distant metastases. Metastasis to the female genital tract from extragenital primaries is comparatively unusual, and when it does occur, the ovaries are the dominant site of involvement, followed by the vagina. The uterus is an uncommon but well-recognised target. Across published series, breast carcinoma accounts for the largest single share of extragenital metastases reaching the uterus, and within the breast cancer population the proportion of patients in whom uterine involvement can be demonstrated has been variously estimated at between 0.8% and 8% depending on the case ascertainment method, the diligence of imaging and pathological review, and whether autopsy data are included [1,2,3].

Two features of breast cancer biology make this metastatic pattern particularly relevant. The first is the disproportionate contribution of invasive lobular carcinoma. Although ILC accounts for only 10–15% of all newly diagnosed breast cancers, it is consistently over-represented among breast cancer metastases to gynaecological organs, peritoneum, retroperitoneum, gastrointestinal tract, and serosal surfaces [4,5]. The mechanistic basis for this distinctive metastatic tropism is closely linked to the loss of E-cadherin function that is the defining molecular event in lobular oncogenesis. Loss of E-cadherin produces the characteristic single-file, discohesive growth pattern of ILC and is associated with a propensity for diffuse, infiltrative spread along stromal planes rather than the formation of discrete mass lesions. This biology has practical consequences both for radiological detection and for histopathological recognition of the metastatic deposit, since lobular metastases often blend into surrounding endometrial or myometrial stroma and may be missed on routine examination.

Second, endocrine therapy plays a central role in the management of hormone receptor-positive breast cancer. Tamoxifen, a selective estrogen receptor modulator, exerts an antagonist effect in breast tissue but a partial agonist effect on the endometrium. Long-term tamoxifen use in breast cancer survivors is therefore associated with an increased incidence of endometrial polyps, hyperplasia, and a measurable but small increase in the risk of primary endometrial carcinoma, particularly in postmenopausal women [6,7,8]. The clinical consequence is that breast cancer survivors on tamoxifen frequently experience postmenopausal bleeding and undergo endometrial sampling, generating a population in whom both primary endometrial pathology and metastatic disease must be considered. Aromatase inhibitors, used in postmenopausal women, do not appear to share this endometrial proliferative effect to the same degree, although metastatic breast cancer to the uterus has been reported in patients on anastrozole, letrozole, and exemestane as well [9,10,11,12].

The clinical importance of recognising uterine metastasis from breast carcinoma rests on three pragmatic considerations. First, treatment differs fundamentally between a primary uterine carcinoma and a metastatic deposit: the former is typically managed with curative-intent surgery and adjuvant therapy directed at uterine disease, while the latter represents disseminated disease for which the principal management is systemic therapy directed at the breast cancer biology, with surgery reserved for symptomatic palliation. Misclassification therefore has direct therapeutic consequences. Second, the histological appearance of metastatic breast carcinoma in the uterus—particularly the discohesive cells of metastatic ILC infiltrating endometrial stroma—can closely mimic primary endometrial pathology including endometrial stromal sarcoma, poorly differentiated adenocarcinoma, and lymphoma, especially in small biopsy specimens. Immunohistochemistry is essential for the correct diagnosis [13,14]. Third, the existence of a small but identifiable subgroup of patients in whom uterine bleeding leads to the discovery of an otherwise occult breast cancer means that the differential diagnosis of postmenopausal bleeding cannot be confined to gynaecological pathology [15,16,17,18].

Despite these considerations, the literature on uterine metastasis from breast carcinoma is fragmented. Most published reports are individual case reports or small case series, and few systematic syntheses have been performed since the foundational clinicopathological studies of Kumar and Hart [1] and Mazur and colleagues [3], which together examined autopsy and surgical material from a previous era. The molecular and therapeutic context of breast cancer has changed substantially in the intervening four decades, and the population now generating uterine metastases is a population of long-survivors on extended endocrine therapy with much higher rates of human epidermal growth factor receptor 2 (HER2) testing and targeted therapy. A contemporary synthesis is therefore warranted.

We compiled and analysed 105 individual cases of uterine metastasis from breast carcinoma reported between 1982 and 2025. To our knowledge this represents the largest single compilation of such cases in the published literature, surpassing the recent systematic analysis by Salati and colleagues [19] which compiled 55 cases reported between 2010 and 2022. The analysis aimed to characterise the histological predilection of the disease, formally test the association between tamoxifen exposure and polyp-substrate metastasis, examine the latency distribution and its implications for long-term surveillance, and define the diagnostic immunohistochemical panel that allows reliable distinction between metastatic breast carcinoma and primary uterine pathology. We sought to translate these patterns into practical recommendations for breast cancer survivors with uterine pathology.

## 2. Materials and Methods

### 2.1. Search Strategy

We performed a structured literature search of PubMed/MEDLINE and Google Scholar, supplemented by hand-searching of the reference lists of identified articles and of recent review articles on the topic. This combination was chosen deliberately, because case reports of rare entities are frequently published in journals not indexed by subscription databases, and complementing PubMed/MEDLINE with Google Scholar and citation tracking is well suited to comprehensive retrieval of the case-level literature. The search covered the period from 1 January 1980 to 19 December 2025 and used combinations of the following terms: “breast cancer”, “breast carcinoma”, “invasive lobular carcinoma”, “invasive ductal carcinoma”, “uterine metastasis”, “endometrial metastasis”, “myometrial metastasis”, “cervical metastasis”, “endometrial polyp”, “tamoxifen”, and “GATA3”. Foreign-language reports were included where an English-language abstract or full-text translation was available; reports identified only by citation in subsequent reviews were included where sufficient case-level data could be extracted from the citing article. The search was iterative, with cross-references identified through the reading of each retrieved article being added to the search pool. The flow of records from initial identification through final inclusion is summarised in a literature search and case selection flow diagram (Supplementary Figure S1). This work is presented as a structured narrative review of the published case literature; it was not pre-registered as a systematic review protocol. Because formal risk-of-bias instruments developed for comparative studies cannot be applied meaningfully to single-patient case reports, the methodological quality of the included literature was instead appraised at the corpus level against the framework of Murad and colleagues for case reports and case series [20], across the domains of selection, ascertainment, causality, and reporting; this appraisal is summarised in Supplementary Table S2. Records were screened and case-level data were extracted independently by two authors, with discrepancies resolved by discussion.

### 2.2. Inclusion and Exclusion Criteria

Cases were included if (i) the primary tumour was a histologically confirmed breast carcinoma; (ii) metastatic deposits were demonstrated histologically within the uterus, defined as the endometrium, myometrium, endometrial polyp, leiomyoma, or cervix; and (iii) the diagnosis of metastasis was supported by either an unequivocal clinical context or by immunohistochemical confirmation of breast origin. Both synchronous (uterine deposit identified at or before the breast diagnosis) and metachronous presentations were eligible. Cases in which only the ovary was involved without uterine involvement were excluded. Pure cervical metastases were included in the uterine dataset, as the differential diagnostic considerations overlap substantially with those of corpus deposits. Cases identified at autopsy were retained where individual case-level data were reported, but the seminal autopsy-derived series of Kumar and Hart [1] and Mazur and colleagues [3] are referenced separately because individual-level data from these series are not all extractable. Where two or more cases were reported in a single article, each was treated as a separate entry where individual data were available.

### 2.3. Data Extraction

For each case the following variables were extracted: patient age at uterine metastasis diagnosis, histological subtype of the breast primary, anatomical site or sites of uterine involvement, dimensions of the uterine deposit where reported, hormone receptor and HER2 status of the primary or metastatic tumour, prior systemic and locoregional therapy received before the uterine metastasis was diagnosed, presenting symptoms or context of detection, latency from primary breast cancer diagnosis to uterine metastasis diagnosis (in months), reported outcome with time to relevant event where available, and stage of the original breast cancer. Where a variable was not reported in the source article it was coded as not available (“na”). Latency was coded as zero for cases in which the uterine deposit was identified within three months of the breast primary diagnosis (synchronous), and as a negative value for the rare cases in which the uterine deposit preceded the recognition of the breast primary by an interval of weeks to years (occult primary).

### 2.4. Data Synthesis

Continuous variables were summarised as median, mean, and range; proportions were calculated using cases with non-missing data as denominators, with the denominator stated for each result. Categorical comparisons (notably polyp-substrate involvement in tamoxifen-exposed versus non-exposed patients) were tested with Fisher’s exact test (two-sided), and odds ratios with 95% CI were calculated. No formal meta-analysis was performed because the underlying case reports do not share common comparator arms or outcomes. Given the rarity of the condition and the case-report-based nature of the dataset, the statistical comparisons reported here should be regarded as descriptive of the case-report literature rather than as inferential of the broader patient population. Statistical analyses were performed using MedCalc Statistical Software (version 22.026, MedCalc Software Ltd., Ostend, Belgium); the final literature search was conducted on 19 December 2025.

### 2.5. Search Yield

To minimise the risk of double-counting individual patients in the compiled dataset, a structured de-duplication procedure was applied. For every retrieved case we cross-checked the following identifiers against all previously included entries: lead and senior author surnames, institution of origin, country, year of breast cancer diagnosis, year of uterine metastasis diagnosis, patient age at uterine metastasis, primary breast tumour histology and stage, and the specific anatomical sites of uterine involvement. Cases sharing two or more of these identifiers were flagged for closer comparison against the full text of the source publications to determine whether the same patient was being reported in more than one venue (for example, as part of an institutional series and subsequently as a detailed individual case report). Where overlap was confirmed, the more detailed and complete entry was retained and the duplicate was excluded. A residual risk of un-detected duplication cannot be entirely eliminated because some early case reports do not provide unique patient identifiers, and we have included this caveat in the Limitations.

From the combined searches we identified 105 individual cases meeting the inclusion criteria from approximately 79 distinct source publications [1,2,3,4,5,6,7,8,9,10,11,12,13,14,15,16,17,18,19,21,22,23,24,25,26,27,28,29,30,31,32,33,34,35,36,37,38,39,40,41,42,43,44,45,46,47,48,49,50,51,52,53,54,55,56,57,58,59,60,61,62,63,64,65,66,67,68,69,70,71,72,73,74,75,76,77,78,79,80,81,82,83,84,85,86,87,88,89,90,91,92,93,94,95,96,97,98,99,100,101,102,103,104,105,106,107,108,109]; eligible reports were published between 1982 and 2025, with no qualifying publications identified within the earlier 1980–1981 portion of the search window. During source verification, one duplicate entry was identified and removed (an entry referring to the same patient as the index case of Franco-Márquez and colleagues [21]), and three additional entries were corrected against their original publications to ensure accurate age, anatomical site, and outcome information. The seminal series of Kumar and Hart [1] and Kumar and Schneider [2] are entered as series-level entries with aggregate descriptors only.

## 3. Results

### 3.1. Patient Demographics

The demographic, histopathological, molecular, and clinical characteristics of the 105 compiled cases are summarised in Table 1. Patient age at the time of uterine metastasis diagnosis was reported in 85 of 105 cases. The mean age was 58.5 years, the median was 58 years, and the range was 32 to 92 years (interquartile range 50 to 66 years). Twenty-one patients (24.7%) were younger than 50 years at the time of uterine metastasis diagnosis, 48 (56.5%) were aged 50–69, and 16 (18.8%) were 70 years or older. The age distribution mirrors that of the underlying breast cancer population reasonably well, with a perhaps slight enrichment of younger patients reflecting the long latency seen in some lobular carcinoma cases. Patients diagnosed with breast cancer in their forties may not develop uterine metastases until their sixties or seventies. The youngest patient was 32 years old at uterine metastasis diagnosis [22] and the oldest was 92 years [23]. The annual case yield has increased substantially across the period covered by this review: 2 cases were reported in the 1980s, 10 in the 1990s, 22 in the 2000s, 36 in the 2010s, and 34 in the 2020s through the search cut-off date. This temporal trend likely reflects a combination of greater diagnostic recognition (driven by routine availability of immunohistochemistry from the late 1990s onwards), the growing population of breast cancer survivors at risk for late recurrence, and increased clinical awareness of this entity.

### 3.2. Histological Subtype

Most prominent among our findings is the disproportionate contribution of invasive lobular carcinoma to the uterine metastatic compartment. Among the 88 cases for which the breast primary was assigned to a specific histological subtype, ILC accounted for 51 cases (58%), invasive ductal carcinoma (IDC; the older WHO designation) or invasive breast carcinoma of no special type (IBC NOS; the current WHO 2019 designation for the same entity) accounted for 32 cases (36%), mixed lobular and ductal carcinoma accounted for two cases (2.2%), invasive micropapillary carcinoma accounted for one case [24], and signet-ring cell variants of the breast accounted for two cases [25,26]. One additional case was reported as a metaplastic breast carcinoma with heterologous mesenchymal differentiation [27]; this case sits within the broader spectrum of rare low-grade triple-negative variants reviewed recently [28]. In this manuscript, both IDC and IBC NOS terms are used where they reflect the terminology of the original source publications; both designations refer to the same WHO entity.

**Figure 1:**
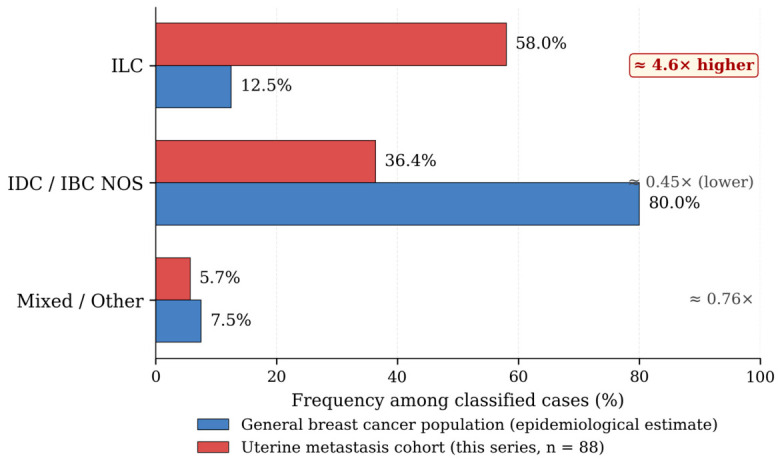
Histological subtype distribution among the uterine metastasis cohort (*n* = 88 classified cases) compared with population frequencies in the general breast cancer population. Invasive lobular carcinoma (ILC) is approximately 4.6× over-represented relative to its population frequency, while invasive ductal carcinoma/invasive breast carcinoma not otherwise specified (IDC/IBC NOS) is correspondingly under-represented. Population frequencies are based on the WHO Classification of Breast Tumours, 5th edition [109], which reports an approximate frequency of 10–15% for ILC and 70–80% for invasive carcinoma of no special type in newly diagnosed invasive breast cancer.

When set against the population frequencies—ILC representing approximately 10–15% of newly diagnosed invasive breast cancers, IDC and IBC NOS together accounting for the majority of the remainder—the over-representation of ILC in the uterine metastatic compartment is approximately four- to six-fold (Figure 1). This pattern holds across the period of publication and across the geographical origin of the reports, suggesting it is not an artefact of evolving diagnostic practice. The biological basis for this over-representation is considered in the Discussion (Section 4.1).

### 3.3. Anatomical Distribution Within the Uterus

**Figure 2:**
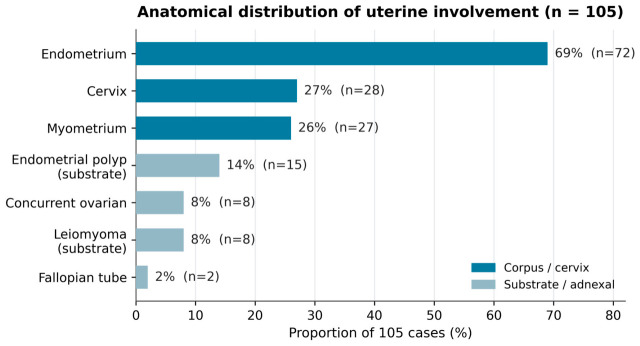
Anatomical distribution of metastatic involvement across the 105 pooled uterine cases.

The anatomical distribution of uterine involvement is summarised as follows. The endometrium was involved in 72 of 105 cases (69%), the myometrium in 27 (26%), and the cervix in 28 (27%) (Figure 2). An endometrial polyp was the specific substrate of metastatic deposition in 15 cases (14%); a uterine leiomyoma was involved in 8 cases (8%) [29,30,110], and one case described metastasis to a uterine lipoleiomyoma [31]. Concurrent ovarian involvement was documented in 8 cases (7.6%), although this proportion is almost certainly underestimated because not all reports specified ovarian status. Fallopian tube involvement was reported in two cases.

Multiple uterine sites were involved in approximately half of the cases for which a clear single-versus-multiple-site assessment could be made. Combinations such as endometrium plus myometrium (in some cases also extending to the cervix or fallopian tube) were the typical pattern of multifocal involvement. Pure cervical metastasis without involvement of the corpus was documented in a smaller subset, with at least 5 well-described cases [32,33,34,35,36]. The radiological appearance in pure cervical cases can closely resemble primary cervical carcinoma, with the differential established only by immunohistochemistry.

Polyp-based metastasis at this frequency deserves comment. The polyp itself is the substrate of the metastatic deposit, with the metastatic deposit either replacing the polypoid stroma or sitting as a microscopic focus within an otherwise benign-appearing polyp. In some reports the metastatic focus measured less than 2 millimetres [18], highlighting the importance of careful histological examination of every polyp removed from a patient with a history of breast cancer.

### 3.4. Molecular Profile

Hormone receptor and HER2 status were reported in a subset of cases, with reporting much more complete in cases published after approximately 2005. To quantify this disparity, HER2 status was reported in only 1 of 22 cases (4.5%) published before 2005, compared with 37 of 83 cases (44.6%) published from 2005 onward (Fisher’s exact test, *p* = 0.0003), confirming that the apparent molecular profile of this literature is shaped substantially by era-dependent ascertainment rather than by a stable biological denominator. Of the 60 cases for which estrogen receptor status was reported, 54 (90%) were ER-positive and 6 (10%) were ER-negative. Of the 38 cases with HER2 status reported, 15 (39%) were HER2-positive and 23 (61%) were HER2-negative. Triple-negative breast cancer was reported in only 3 cases [25,37,38], all of which were unusual individual reports because of the histology rather than because triple-negative disease is over-represented in this metastatic pattern.

ER positivity at 90% in reported cases substantially exceeds the population rate of ER-positivity in newly diagnosed breast cancer (approximately 70–75%), and likely reflects a combination of three factors. First, ER-positive disease has the long natural history that allows late recurrences to manifest clinically as uterine metastasis, sometimes decades after the primary. Second, ER-positive disease is the population in which tamoxifen is most often used, and tamoxifen-associated endometrial pathology generates the diagnostic encounters that bring uterine metastasis to clinical attention. Third, ILC is overwhelmingly an ER-positive tumour, and the ILC enrichment of this metastatic compartment carries a corresponding ER enrichment with it. The HER2-positivity rate of approximately 40% is also higher than the contemporary population rate of approximately 15–20%, and this excess merits explicit interpretation. Three non-exclusive mechanisms are plausible. First, HER2 testing was not routinely performed in the older cases included in this synthesis, so the cases in which HER2 status is reported are biased toward the more recent era in which HER2-directed therapy was available; cases with documented HER2 positivity are more likely to be reported because the molecular profile is itself a publishable finding linked to specific therapy. Second, HER2-positive disease in the era before targeted therapy was characterised by an aggressive metastatic course, and a subset of patients now reaching the uterine compartment as long-survivors have done so under HER2-directed therapy that prolonged their disease course; the population generating contemporary uterine metastasis cases therefore contains a higher fraction of HER2-positive patients than the population at original diagnosis. Third, true biological enrichment cannot be excluded, but the case-report nature of the dataset does not allow this to be tested. The ~40% rate should therefore be interpreted as a description of HER2 prevalence in the published case literature rather than as a population-level estimate of HER2 prevalence in uterine breast metastases.

### 3.5. Prior Systemic and Locoregional Therapy

Tamoxifen exposure was documented in 41 of 105 patients (39%), aromatase inhibitor exposure in 12 (11%), and any endocrine therapy in 58 (55%). Cytotoxic chemotherapy had been administered in approximately 35 cases (33.3%), and at least 18 cases (17.1%) had received locoregional radiotherapy to the breast or chest wall. Approximately 12 cases (11.4%) had received no prior systemic or locoregional therapy at the time the uterine metastasis was identified, generally because the uterine deposit was synchronous with or preceded the diagnosis of the breast primary.

#### 3.5.1. The Tamoxifen–Polyp Association

A particular pattern emerges when tamoxifen exposure is cross-tabulated against the type of uterine substrate involved. Among the 41 patients with documented tamoxifen exposure, 12 had metastatic deposits located within an endometrial polyp (29%). Among the 64 patients without documented tamoxifen exposure, only 3 had polyp-based deposits (5%). The “non-exposed” group as defined here includes both patients in whom tamoxifen was explicitly stated to have not been administered and patients in whom tamoxifen status was simply not reported in the source publication; we have treated absence of mention as absence of exposure on the conservative grounds that tamoxifen-relevant cases tend to have tamoxifen exposure explicitly stated by reporting authors, but this convention introduces a potential misclassification bias that we revisit in the Limitations. The association is statistically significant (Fisher’s exact test, two-sided *p* = 0.0009); the crude odds ratio for polyp-substrate metastasis in tamoxifen-exposed compared with non-exposed patients is 8.41 (95% CI: 2.20–32.14), and the corresponding risk ratio is 6.24 (95% CI: 1.88–20.79). The wide upper bound of the confidence interval reflects the small absolute number of polyp events, but the lower bound comfortably excludes unity and the absolute risk difference of 24.6 percentage points (29.3% versus 4.7%) is clinically substantial. The signal is biologically coherent: tamoxifen is a well-established cause of endometrial polyps [7,39], and the polyp tissue may serve as a permissive site for the implantation and growth of circulating tumour cells through mechanisms that include disturbed local angiogenesis and altered stromal–epithelial interactions in the polyp microenvironment. The clinical inference is that polyps removed from women on tamoxifen who have a history of breast cancer warrant careful histological examination with a low threshold for ancillary immunohistochemistry, particularly when the morphology is unusual [23,40,41,42,43,44].

Two sensitivity analyses confirm the robustness of this signal. First, restricting the non-exposed group to the 42 patients in whom tamoxifen was explicitly reported as not administered (excluding the 22 patients with unreported status) yielded a more conservative odds ratio of 5.38 (95% CI: 1.39–20.82, *p* = 0.011), demonstrating that the association is not an artefact of misclassifying unreported cases as non-exposed. Second, analysis restricted to the 92 cases published from 2000 onwards (87.6% of the cohort)—the period during which contemporary immunohistochemical and molecular profiling became routinely available—produced essentially unchanged estimates: ILC frequency 60.0% (vs. 58.4% overall), tamoxifen–polyp OR 7.33 (95% CI: 1.88–28.59, *p* = 0.002) in the primary analysis and 5.22 (95% CI: 1.32–20.57, *p* = 0.017) in the sensitivity analysis. The full set of estimates is presented graphically in Supplementary Figure S2; all four confidence intervals exclude OR = 1, supporting a consistent and robust association across the analyses. The principal findings of this synthesis are therefore not driven by older or under-characterised reports.

#### 3.5.2. Comparison of ILC and IDC/IBC NOS Subgroups

To examine whether the polyp signal observed in our overall analysis is driven by histological subtype rather than by tamoxifen exposure, we compared anatomical and treatment variables between the ILC and IDC/IBC NOS subgroups (Table 2). Within the uterine compartment, anatomical distributions were largely similar between the two histological groups: endometrial, myometrial, cervical, and ovarian involvement did not differ significantly. Polyp-substrate involvement showed a numerical excess in the ILC subgroup (21.6% vs. 9.4%, OR 2.66, 95% CI 0.68–10.4) but did not reach statistical significance (Fisher’s *p* = 0.23), and tamoxifen exposure was very similar in both groups (47.1% vs. 40.6%, *p* = 0.65). These subgroup comparisons should be interpreted with caution given the small absolute event counts within each cell, particularly for polyp-substrate involvement, which limits the statistical power to detect modest differences. Accordingly, the absence of statistically significant differences between subgroups should not be interpreted as evidence that the subgroups are equivalent. The principal interpretive implication is that the strong tamoxifen–polyp association demonstrated above (OR 8.41) is driven primarily by tamoxifen exposure rather than by histological subtype, although a smaller histology-driven contribution cannot be excluded with the available sample size. The over-representation of ILC in the uterine metastatic compartment therefore appears to be determined predominantly at the level of selection for arrival at the uterus (i.e., which breast cancers metastasise here at all) rather than at the level of where they implant once they arrive.

### 3.6. Presenting Symptoms and Context of Detection

Of the 91 cases in which the presenting context was specified, abnormal uterine or vaginal bleeding was the dominant symptom, present in 62 cases (68%). The bleeding was variably described as postmenopausal bleeding, menorrhagia, metrorrhagia, intermenstrual bleeding, or simply abnormal vaginal bleeding. Pelvic or abdominal pain, abdominal distension, or a palpable mass accounted for a further 8 cases (8.8%), and miscellaneous symptoms including urinary frequency [24] and an exteriorised vaginal lump [45] accounted for 2 additional cases. Notably, 18 of 91 patients (20%) were entirely asymptomatic at the time of uterine metastasis detection. Asymptomatic detection occurred most often through positron emission tomography or computed tomography surveillance for breast cancer follow-up [12,46,47,48,49], less commonly through routine gynaecological surveillance, and occasionally through transvaginal ultrasound performed for an unrelated indication or through cervical cytology.

An asymptomatic presentation rate of 21% is clinically meaningful. It indicates that uterine metastasis is not always heralded by bleeding and that imaging-based detection (now common with routine surveillance imaging in breast cancer survivors) accounts for a substantial fraction of contemporary diagnoses. This has implications for the differential diagnosis of incidental uterine findings on positron emission tomography–computed tomography (PET-CT) or other cross-sectional imaging in this population.

### 3.7. Latency from Primary to Uterine Metastasis

The latency from breast cancer diagnosis to identification of uterine metastasis was reported in 74 cases. The median latency was 36 months and the mean was 54.9 months, with a range from −24 months (uterine metastasis identified before the breast primary) to 360 months (30 years after the primary). Thirteen cases (17.6%) were synchronous, defined as uterine and breast involvement identified within three months of one another. At least one case in this compilation [18] and arguably two further cases [15,44] had the uterine deposit identified before the breast primary was recognised, with the breast tumour discovered through subsequent dedicated workup. Four cases (5.4%) had a latency between 3 and 12 months. Thirty-one cases (41.9%) had a latency between 1 and 5 years, 18 cases (24.3%) between 5 and 10 years, and 8 cases (10.8%) had a latency exceeding 10 years.

**Figure 3:**
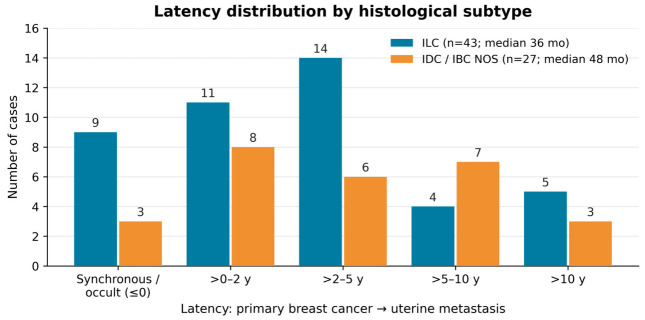
Latency from breast cancer diagnosis to detection of uterine metastasis, stratified by histological subtype (ILC versus IDC/IBC NOS).

Delayed recurrence in breast cancer becomes particularly evident when latency is stratified by histological subtype. For ILC the median latency was 36 months and the mean 58.0 months, with a maximum of 360 months. For IDC and IBC NOS the median was 48 months and the mean 54.7 months, with a maximum of 240 months (Figure 3). The longest latencies—Franco-Marquez and colleagues 30 years [21], Awazu and colleagues 23 years [50], Dye and colleagues 20 years [36], Trihia and colleagues 19 years [51]—almost all fall within the lobular carcinoma group. The biological correlate is the well-recognised propensity of ILC for late recurrence, attributed to the slower proliferative kinetics of lobular tumour cells and to the indolent micrometastatic behaviour conferred by E-cadherin loss.

### 3.8. Outcomes

Outcome data of any kind were reported in 29 of 105 cases. Among these 29 cases, 13 patients (44.8%) died of disease at last reported follow-up, 13 patients (44.8%) were alive with reported progression-free survival or disease-free interval, two patients had documented disease progression but were not reported deceased, and one was lost to follow-up. The median time from uterine metastasis diagnosis to death in those who died was 6 months (mean 8 months, interquartile range 4–11 months, total range 2–24 months). When stratified by histological subtype, the small numbers preclude meaningful comparison: ILC patients with reported death had a median survival of 6 months (*n* = 7) and IDC/NOS patients had a median survival of 4 months (*n* = 4). Among the 13 patients reported as alive at last follow-up, the median follow-up was 12 months (range 6–32 months). These figures must be interpreted in the context of severe reporting bias: only a minority of cases include any follow-up information, and cases with long survival are systematically more likely to be reported because the authors have time to write them up. The 6-month median survival in the death subgroup is therefore consistent with rapid progression in patients in whom uterine metastasis is part of widespread disease, but is not a reliable estimator of overall prognosis for the cohort. The increasing representation of survivors in cases published after approximately 2017 likely reflects the introduction of effective targeted therapies (cyclin-dependent kinase 4/6 [CDK4/6] inhibitors combined with endocrine therapy, and dual HER2 blockade) for metastatic breast cancer.

### 3.9. Special Subgroups

#### 3.9.1. The Occult Primary Subgroup

A small but distinctive subgroup of patients presented with uterine pathology that led to the diagnosis of a previously unsuspected breast cancer. This subgroup includes the recent case of Rousselot and colleagues [18], in which an incidental microscopic focus of metastatic ILC was identified in an endometrial polyp removed for postmenopausal bleeding from a woman with no breast symptoms; the identification of metastatic ILC prompted a dedicated breast workup that demonstrated the primary tumour two years after the polyp diagnosis. The case of Cimpeanu and colleagues [17] involved a 76-year-old woman whose presenting complaint was hip pain and whose subsequent workup identified a cervical lesion that immunohistochemistry assigned to a breast primary, with the breast itself initially showing no abnormality on imaging. Earlier, the case of Bogliolo and colleagues [16] described a 78-year-old woman in whom routine gynaecological examination disclosed a markedly enlarged cervix and uterine body that proved on biopsy to be metastatic lobular breast carcinoma; the breast tumour was identified subsequently and was small. The Taxy and Trujillo case [15] also describes endometrial metastasis as the initial manifestation of breast cancer. The clinical principle that emerges from this subgroup is that metastatic breast carcinoma must figure in the differential diagnosis of unexplained postmenopausal uterine bleeding even in women without a known breast cancer history, particularly when the histology shows discohesive single cells, signet-ring forms, or otherwise atypical morphology in an endometrial sample.

#### 3.9.2. The Synchronous Presentation Subgroup

Distinct from the occult primary cases above, twelve cases in this series were synchronous in the sense that the breast and uterine diagnoses were established within three months of one another. In these cases the breast cancer was usually known and the uterine deposit was identified as part of staging or in response to early gynaecological symptoms. Synchronous presentations were enriched for advanced breast cancer at primary diagnosis (T3 or T4 disease, multiple positive nodes), and several were associated with HER2-positive disease [34,52,53].

#### 3.9.3. The Very-Late Recurrence Subgroup

Eight patients had a latency exceeding 10 years from primary breast cancer diagnosis to uterine metastasis. The longest interval was 30 years [21], with multiple cases at 19–23 years [36,50,51], and a cluster at 11–14 years [23,54,55]. The lobular histology dominates this subgroup almost exclusively, consistent with the indolent biology of metastatic ILC. Practically, this means that gynaecological symptoms in a woman with a remote history of lobular breast cancer should not be dismissed as unrelated to the breast diagnosis simply because of the time interval.

#### 3.9.4. The Asymptomatic Detection Subgroup

Asymptomatic detection was documented in 18 cases (19.8% of those with the presenting context specified). The detection routes included PET-CT imaging performed for breast cancer surveillance [12,47,48,49], routine gynaecological examination [16,56], screening cytology [52], and incidental findings on imaging performed for other indications [53]. In the present series, the proportion of asymptomatic detections was numerically higher among cases published after 2015 than before (22% versus 16%), although this difference did not reach statistical significance (Fisher’s exact test, *p* = 0.6). Any such trend, if real, would most plausibly reflect the more widespread use of cross-sectional and PET-CT imaging in metastatic breast cancer surveillance and a gradual recognition of uterine metastasis as an entity to be considered when interpreting surveillance imaging.

## 4. Discussion

These 105 compiled cases permit several conclusions about uterine metastasis from breast carcinoma that are stronger than the conclusions available from any single case report or smaller case series. We summarise these in turn, and then turn to the practical clinical implications and the limitations of the synthesis.

### 4.1. The Biology of Lobular Tropism

Invasive lobular carcinoma accounting for approximately 58% of histologically classified cases is the dominant signal in our compilation. We interpret this finding with caution: morphologically distinctive cases (single-file ILC, signet-ring variants) are more readily recognised and published than conventional NOS metastases, and the over-representation observed here therefore reflects the published case literature rather than a directly measured population frequency. Even allowing for this ascertainment effect, however, the magnitude of the over-representation (approximately five-fold relative to the ILC fraction in the general breast cancer population) is consistent with the well-documented predilection of lobular carcinoma for gynaecological, peritoneal, and gastrointestinal metastasis [4,5] and supports a genuine, biologically grounded tropism that would benefit from confirmation in population-based registry data.

The mechanistic basis is now better understood than at the time of the foundational Kumar and Hart report [1]. The defining molecular event in lobular oncogenesis is loss of E-cadherin function through bi-allelic CDH1 inactivation (mutation on one allele, loss of heterozygosity at 16q22.1 on the other), disrupting homotypic cell adhesion and producing the discohesive single-file growth pattern that is the histological signature of ILC. The same biology that allows the primary tumour to grow as scattered single cells permits efficient intravasation and extravasation, transit through stromal planes without forming a cohesive nodule, and re-implantation in distant sites that include the gastrointestinal tract serosa, the peritoneum, the meninges, and the gynaecological organs.

Within the gynaecological compartment, the endometrial and myometrial stroma offers a hospitable substrate for this single-file infiltrative growth. The metastatic deposit characteristically blends into the surrounding stroma rather than displacing it, and may be visible only as subtle cellularity changes on low-magnification examination. The recent case of Riedinger and colleagues [57], in which a documented CDH1 mutation in the metastatic ILC was associated with peritoneal, myometrial, and cervical disease, provides a contemporary example of the molecular signature corresponding to the histological pattern. The same case is also notable for the coexistence of a primary uterine carcinosarcoma—an instance in which the patient had two distinct synchronous uterine pathologies, both eligible for distinct molecularly directed therapies, and a clear illustration of why correct attribution matters.

### 4.2. Tamoxifen, Endometrial Polyps, and the Implantation Hypothesis

The substantial and statistically robust difference in polyp-substrate prevalence between tamoxifen-exposed and non-exposed patients (29.3% vs. 4.7%; OR 8.41, 95% CI 2.20–32.14; Fisher’s exact *p* = 0.0009) is one of the cleanest signals in this synthesis. The mechanism most consistent with this observation is that tamoxifen-associated endometrial polyps act as a permissive niche for the implantation of circulating breast tumour cells. Tamoxifen exerts a partial agonist effect on the endometrium and is well established as a cause of endometrial polyps, particularly in postmenopausal women who have been on tamoxifen for several years [6,7,39]. The polyp microenvironment is characterised by altered angiogenesis, oedematous stroma, and disturbed epithelial-stromal signalling, all of which may favour the survival of disseminated tumour cells that arrive at the polyp through systemic circulation. An alternative or contributory mechanism is that tamoxifen-treated patients are systematically more likely to undergo gynaecological surveillance and endometrial sampling because of the increased incidence of bleeding from tamoxifen-related endometrial pathology, leading to ascertainment bias in favour of polyp-based diagnoses. This ascertainment effect is important and may be substantial: women receiving tamoxifen undergo heightened gynaecological vigilance, so polyps—and any metastatic deposits within them—are more likely to be sampled, detected, and reported in this group than in women not receiving tamoxifen. The magnitude of the observed association (OR 8.41) should therefore be regarded as an upper estimate that very probably overstates any true biological predilection, and the implantation account above is best read as a biologically plausible hypothesis rather than a demonstrated causal mechanism. Case-level literature of this kind cannot separate a genuine tissue tropism from differential detection, so the association is best treated as hypothesis-generating. Both mechanisms are likely to be operative, and both have practical implications: patients on tamoxifen require careful gynaecological surveillance, and any polyp removed from a patient with a history of breast cancer should undergo careful histological examination with consideration of immunohistochemistry.

Data on aromatase inhibitor-associated uterine metastasis are sparser. Cases reported during anastrozole therapy [9,10,47], letrozole therapy [11,12,50], exemestane therapy, and combinations of these agents make clear that aromatase inhibitor therapy does not protect against uterine metastasis, although the polyp-based pattern is much less prominent in this subgroup. This is consistent with the biology: aromatase inhibitors do not induce endometrial polyps and may even reduce the polyp burden of patients switched from tamoxifen [8], but they cannot interrupt the systemic dissemination of disseminated tumour cells from established micrometastatic disease.

### 4.3. Diagnostic Pitfalls and the Immunohistochemistry of Uterine Breast Metastasis

Diagnostic challenges posed by uterine metastasis from breast carcinoma are most acute on small biopsy specimens, especially curettage material from a patient with postmenopausal bleeding. The differential diagnosis on the histology bench includes primary endometrial adenocarcinoma (especially serous and undifferentiated subtypes), endometrial stromal sarcoma, undifferentiated uterine sarcoma, lymphoma, and rarely a primary cervical adenocarcinoma when the deposit is at the lower uterine segment. Each of these primaries has distinct treatment implications, making the correct attribution clinically critical.

Immunohistochemistry is central to the differential diagnosis. The contemporary consensus immunohistochemical panel for the workup of an unusual uterine epithelial lesion in a patient with possible breast cancer history includes GATA3 (a sensitive marker of breast and urothelial origin, expressed in approximately 80% of metastatic breast carcinomas) [14], TRPS1 (a more recently characterised nuclear transcription factor with high sensitivity across breast carcinoma subtypes, including triple-negative and lobular tumours, and useful as a complement to GATA3 where the latter is weak or negative) [58], mammaglobin (a more specific but less sensitive marker, typically positive in approximately 50–70% of metastatic breast carcinomas), gross cystic disease fluid protein 15 (GCDFP-15; similar specificity, around 50% sensitivity), estrogen receptor and progesterone receptor (positive in the great majority of breast metastases but also in primary endometrioid carcinomas, so non-discriminating in isolation), and paired-box gene 8 (PAX8; positive in primary uterine and ovarian malignancies but negative in breast carcinoma).

A typical workup will combine GATA3 and/or TRPS1 with PAX8: a GATA3-positive and/or TRPS1-positive, PAX8-negative tumour favours breast origin, whereas the converse pattern favours a primary uterine or ovarian malignancy. E-cadherin staining is useful when the histology suggests lobular morphology: loss of E-cadherin supports lobular origin and is informative both about histological subtype and, when a lobular primary is known, about metastatic origin.

**Figure 4:**
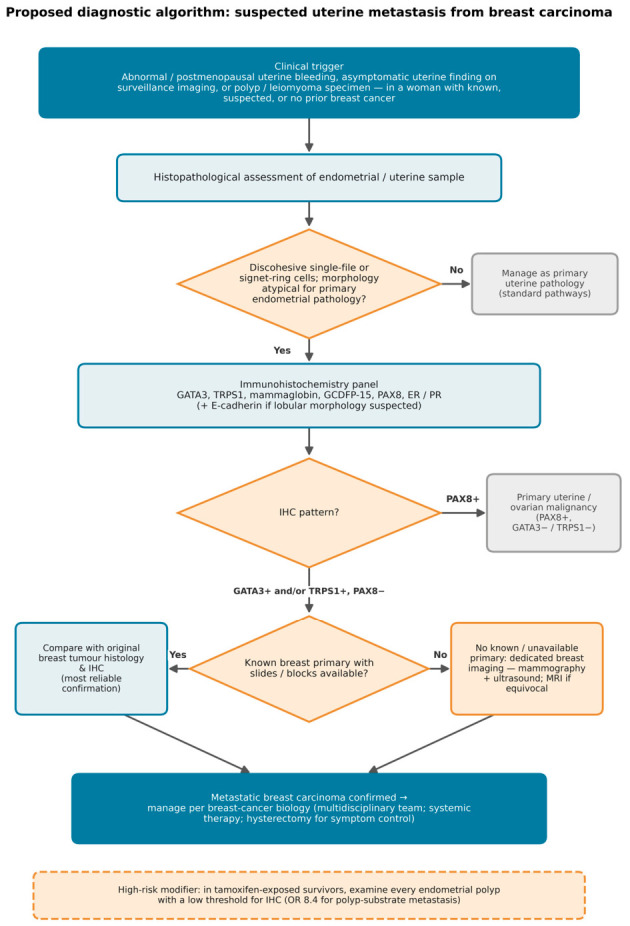
Proposed diagnostic algorithm for the workup of suspected uterine metastasis from breast carcinoma. The pathway begins with the clinical trigger (postmenopausal bleeding, asymptomatic uterine finding, or polypectomy specimen in a woman with known or suspected breast cancer), proceeds through histopathological assessment of the endometrial sample, and uses a combined immunohistochemical panel (GATA3, TRPS1, mammaglobin, GCDFP-15, ER, PR, PAX8, and E-cadherin where lobular morphology is suspected) to distinguish metastatic breast carcinoma from primary uterine pathology. The high-risk modifier callout reflects the strong polyp-substrate association in tamoxifen-exposed patients (OR 8.41, 95% CI 2.20–32.14) demonstrated in the present synthesis. Arrows indicate the direction of the diagnostic workflow; the decision branches are labelled directly on the figure (Yes/No and the immunohistochemical patterns shown), and the arrow (→) in the final box denotes ‘leads to’.

The work of Onuma and colleagues [13] is a cautionary note on the panel: mammaglobin can be expressed in benign and neoplastic endocervical and endometrial tissues and is not infallibly specific for breast origin. A similar caveat applies to TRPS1, which, despite its very high sensitivity for breast carcinoma, has been documented in a substantial proportion of endometrial and ovarian carcinomas and therefore cannot be used in isolation to confirm breast origin in a uterine specimen [58]. The diagnosis therefore rests on the combined pattern of staining, the morphology of the deposit, and the clinical context, rather than on any single marker. Comparison with the original breast tumour histology and immunohistochemistry, where the slides or paraffin blocks are available, is the most reliable confirmatory step and should be sought wherever possible. The principal differential diagnostic entities, their characteristic morphological features, and the immunohistochemical profiles most useful in distinguishing them are summarised in Table 3, and the diagnostic workflow that emerges from these considerations is summarised as a clinical algorithm in Figure 4. This proposed algorithm reflects the patterns identified in the present synthesis together with established diagnostic practice; it is intended as a pragmatic aid and has not been prospectively validated.

### 4.4. Special Clinical Scenarios

Several of the special subgroups identified in the Results merit closer practical discussion. The occult primary scenario—uterine metastasis preceding or coinciding with the recognition of an unsuspected breast tumour—is rare but real [15,16,17,18]. The implication is that breast imaging (mammography and breast ultrasound, with magnetic resonance imaging if those are equivocal) should be considered in any postmenopausal woman with unexplained uterine bleeding in whom the histology shows discohesive single cells, signet-ring forms, or other features atypical for primary endometrial pathology. This is particularly important when the differential diagnosis includes endometrial stromal sarcoma, since the histological mimicry between metastatic ILC and endometrial stromal sarcoma is well documented and the treatments are completely different.

Very-late recurrence reinforces a separate but related clinical principle. A woman with a remote history of ILC who develops gynaecological symptoms decades after her primary diagnosis is not, on the basis of the time interval alone, in a low-pre-test-probability situation. Several cases in this dataset were diagnosed 20 to 30 years after the breast primary [21,36,50,51], and the lobular biology of late recurrence is a well-recognised feature of this disease. Clinicians caring for breast cancer survivors should counsel them that breast cancer surveillance appropriately remains relevant well beyond the conventional five- or ten-year disease-free interval, particularly for patients with lobular histology.

Asymptomatic detection, now common with frequent surveillance imaging, has its own practical implications. An incidental endometrial thickening or uterine mass on PET-CT or computed tomography in a breast cancer survivor warrants careful clinical and radiological correlation, with histological assessment considered when imaging characteristics are atypical or when there is fluorodeoxyglucose avidity. The threshold for tissue sampling should be low in this clinical context [46,59].

### 4.5. Management Considerations

Definitive treatment recommendations for uterine metastasis from breast carcinoma cannot be derived from a case series synthesis, and randomised trial data are unlikely ever to be available given the rarity of the condition. The reported treatment patterns nonetheless provide useful orientation. Across the cases reviewed, total abdominal hysterectomy with bilateral salpingo-oophorectomy was performed in the majority of cases in which surgery was undertaken, generally for control of bleeding or pelvic pain rather than with curative intent [60]. Systemic therapy, directed by the molecular profile of the breast primary and the metastatic deposit, formed the basis of management in most cases. Where systemic therapy is indicated, current international guidelines for the management of metastatic breast cancer should direct treatment selection [111].

Contemporary practice differs substantively from that documented in earlier reports in the dataset. For hormone receptor-positive disease, the combination of a CDK4/6 inhibitor (palbociclib, ribociclib, or abemaciclib) with endocrine therapy is now standard first-line, and ESR1-mutation testing in cases of endocrine resistance may guide the use of newer selective estrogen receptor degraders such as elacestrant. For HER2-positive disease, the antibody-drug conjugate trastuzumab deruxtecan has demonstrated activity against both HER2-positive and HER2-low tumours; given that a substantial fraction of breast carcinomas previously classified as HER2-negative are now reclassified as HER2-low and become eligible for this agent, careful HER2 immunohistochemical assessment (including the 1+ category) is clinically actionable on the metastatic deposit. For ILC specifically, the lobular histology has been associated with a distinctive responsiveness profile to CDK4/6 inhibition and an emerging role for ctDNA monitoring of CDH1 alterations in the metachronous setting.

Whether hysterectomy serves a therapeutic rather than purely palliative role is contested. In the small subset of patients in whom the uterine deposit is the only or the dominant site of disease (sometimes called “oligometastatic” presentations), surgical clearance combined with systemic therapy may achieve durable disease control. Several long-survivor cases in this case series [17,18,56] illustrate that durable responses are achievable in carefully selected patients, although the proportion of all uterine metastasis patients who fall into this oligometastatic subgroup is likely small. In the majority of cases the uterine deposit is one component of disseminated disease and management is dictated by the overall metastatic burden; in this setting, hysterectomy is appropriate primarily for symptom control.

### 4.6. Limitations

Several limitations of this review should be acknowledged. The fundamental limitation is that this case compilation is built from individual case reports and small case series, with all the publication and ascertainment biases that such literature carries. The reporting of variables such as outcome, latency, and molecular profile is incomplete in a substantial fraction of cases, and the missingness is not random. Older cases systematically lack HER2 and molecular profile information because the techniques were not in routine use at the time, and many cases lack outcome information because they were published as snapshots without follow-up. Quantitative estimates derived from this dataset must therefore be interpreted as descriptions of the published case literature rather than as reliable estimates of population-level parameters.

Several specific publication-bias mechanisms warrant explicit acknowledgement because they directly affect the key quantitative findings. First, cases with unusual morphology—particularly invasive lobular carcinoma with its discohesive single-file growth pattern—are more pedagogically interesting and therefore more publishable than morphologically conventional metastases of no special type; the 58.0% ILC frequency reported here is therefore likely to over-estimate the true ILC proportion in the underlying population. Second, cases involving tamoxifen exposure are likely to be over-represented in the published literature because tamoxifen-related endometrial pathology is a well-established teaching topic, and any unusual lesion in a tamoxifen-exposed patient is more likely to be reported; in addition, the denominator of “non-exposed” patients (*n* = 64) was constructed by treating cases without explicit documentation of tamoxifen administration as non-exposed, and any residual misclassification of unrecognised tamoxifen-exposed cases as non-exposed would bias the observed odds ratio toward the null—the headline estimate should therefore be regarded as a lower bound under this assumption. Third, cases with very long latency intervals between the primary breast cancer and the uterine metastasis are intrinsically more publishable than cases with short latency, biasing the upper tail of the latency distribution upward. These mechanisms do not invalidate the qualitative patterns described in this synthesis but reinforce the need to interpret all numerical estimates as describing the case-report literature rather than the population of patients with uterine breast metastases.

A second limitation is the heterogeneous quality of the case-level data. Some reports include detailed immunohistochemistry, molecular characterisation, and treatment history; others provide only the briefest clinical sketch. In compiling these cases we made conservative judgments about ambiguous variables, and where a report did not specify a particular variable we coded it as not available rather than imputing. The series-level entries from the 1980s [1,2,3] contribute additional cases to the broader literature but do not provide individual-level data extractable for case-level analysis, and these cases are referenced separately rather than included as individual entries. The foundational autopsy-based series of Kumar and Hart [1] identified uterine involvement in a meaningful fraction of breast cancer deaths, providing an estimate from a non-selected post-mortem population that is conceptually complementary to ours; our case-report-based prevalence cannot be directly compared with their autopsy-based prevalence because the two denominators (published case reports vs. all breast cancer deaths) are fundamentally different. Where individual-level data could be extracted from these series, the cases are included. Although a structured de-duplication procedure was applied (Section 2.5), some residual risk of un-detected double-counting cannot be entirely excluded because early case reports occasionally lack the unique patient identifiers (precise institution, patient age, and treatment timeline) needed to confirm overlap with certainty.

A third limitation concerns geographical and language coverage. The search was conducted in English, and although foreign-language reports were included where English-language abstracts or translations were available, this collection is likely to under-represent reports published in languages and journals outside the major bibliographic databases. The retrospective series of Rodrigues and colleagues [61] was identified by citation but the individual cases could not be extracted because the full text was not accessible during the period of this review.

Finally, the rapidly evolving treatment options for metastatic breast cancer mean that the prognostic and therapeutic conclusions drawn from these cases must be interpreted as reflecting the era in which the cases were reported. The introduction of CDK4/6 inhibitors (palbociclib, ribociclib, abemaciclib) for hormone receptor-positive disease, of trastuzumab deruxtecan and tucatinib for HER2-positive disease, and of immune checkpoint inhibitors and PARP inhibitors for selected molecular subgroups during the past decade has substantially improved the outlook for metastatic breast cancer in general, and these advances are unevenly represented across the temporal range of the dataset. A related limitation is that the HER2-low category (defined as immunohistochemistry score 1+ or 2+ with negative in situ hybridisation) cannot be reliably reconstructed retrospectively from the source case reports: most older reports record HER2 only as positive or negative without the granular 0/1+/2+ scoring needed to identify HER2-low cases. The fraction of patients in our cohort who would today qualify for trastuzumab deruxtecan on HER2-low grounds therefore cannot be estimated from these data.

### 4.7. Future Research Directions

Several specific research directions emerge from this synthesis. A prospective multicentre registry of biopsy-confirmed gynaecological tract metastases in breast cancer survivors, with standardised collection of histological subtype, molecular profile, prior endocrine and cytotoxic therapy, anatomical pattern of involvement, and follow-up, would provide a more reliable estimate of incidence and survival than the published case literature can support. Such a registry would also allow formal testing of the polyp-substrate hypothesis in a population-based denominator rather than the biased denominator of cases that reach publication.

Comparative molecular profiling of paired primary breast and metastatic uterine specimens is a second priority. The few cases in which paired sequencing has been performed [57] have illustrated that the metastatic clone may carry additional genomic events relative to the primary tumour, and a larger comparative series would clarify the role of CDH1 mutation status, ESR1 mutation acquisition under endocrine pressure, and other clonal evolution events in driving the metastatic phenotype. The biology of the polyp microenvironment as a permissive niche for circulating tumour cell implantation is also amenable to direct study using surgical specimens, organoid models, and circulating tumour cell capture in tamoxifen-exposed and non-exposed breast cancer cohorts.

Three operational questions emerging from this synthesis remain underexplored: (i) the optimal endometrial sampling strategy for tamoxifen-exposed breast cancer survivors with postmenopausal bleeding, and whether any subgroup warrants routine surveillance hysteroscopy in the absence of symptoms; (ii) whether hysterectomy with bilateral salpingo-oophorectomy provides a survival advantage over systemic therapy alone in apparently isolated uterine metastasis; and (iii) whether a clinically usable risk score can be developed to identify survivors at elevated risk of uterine metastasis on the basis of histology, receptor status, prior tamoxifen exposure, and time since diagnosis. Each is tractable with a sufficiently large prospective cohort.

The principal clinical and diagnostic messages of this review are summarised in Table 4.

## 5. Conclusions

Uterine metastasis from breast carcinoma is rare but well-described, and the compilation of 105 individual cases reported between 1982 and 2025 supports five clinically actionable conclusions. These conclusions are drawn from the published case literature rather than from population-based data; the proportions cited throughout should accordingly be read as descriptive of reported cases, not as the true prevalence of uterine metastasis in the breast cancer population.

First, invasive lobular carcinoma is the dominant histological subtype, accounting for approximately three-fifths of cases despite representing only one-tenth to one-seventh of the underlying breast cancer population. The roughly five-fold over-representation reflects the distinctive E-cadherin-driven discohesive biology of lobular carcinoma and means that a uterine deposit with single-file or signet-ring morphology should raise immediate suspicion of breast origin even when no primary is yet known.

Second, tamoxifen-associated endometrial polyps are a high-risk substrate for metastatic deposition. The strong and statistically robust association observed here (odds ratio 8.41, 95% CI 2.20–32.14; Fisher’s exact *p* = 0.0009) means that any polyp removed from a tamoxifen-exposed breast cancer survivor should be examined with active consideration of the differential diagnosis of metastatic breast carcinoma.

Third, immunohistochemistry is the diagnostic backbone. A combined panel of GATA3, TRPS1, mammaglobin, GCDFP-15, hormone receptors, PAX8, and (for lobular morphology) E-cadherin distinguishes metastatic breast carcinoma from primary uterine pathology in the majority of cases. Where slides or paraffin blocks of the original breast tumour are available, direct comparison is the most reliable confirmatory step.

Fourth, recurrence may occur decades after the breast primary, with very-late presentations (greater than ten years) documented in eight cases in this series and individual reports extending to thirty years. These findings support continued long-term clinical awareness of the possibility of late recurrence in breast cancer survivors, particularly those with lobular histology.

And fifth, approximately one in five patients is asymptomatic at uterine metastasis detection, with the diagnosis made through surveillance imaging rather than clinical symptoms. Incidental uterine findings in breast cancer survivors warrant careful clinical and radiological correlation, with histological assessment considered when imaging characteristics are atypical or when the clinical context raises suspicion of metastatic involvement.

Taken together, these patterns argue for a low threshold for endometrial sampling in breast cancer survivors with abnormal uterine bleeding (particularly on tamoxifen) and for thoughtful immunohistochemical workup of any unusual uterine pathology in this population.

## Figures and Tables

**Table 1:** Demographic, histopathological, molecular, and clinical characteristics of 105 compiled cases of uterine corpus metastasis from breast carcinoma reported in the published literature, 1982–2025.

Characteristic	Value
Demographics	
Total cases	105
Age at uterine metastasis (years; *n* = 85 reported)	
Mean (SD)	58.5 (±12.7)
Median (range)	58 (32–92)
Interquartile range	50–66
<50 years, *n* (%)	21 (25%)
50–69 years, *n* (%)	48 (56%)
≥70 years, *n* (%)	16 (19%)
Breast cancer histology, *n* (%) ^1^	
Invasive lobular carcinoma (ILC)	51 (58%)
Invasive ductal carcinoma/IBC NOS	32 (36%)
Mixed lobular and ductal	2 (2%)
Invasive micropapillary carcinoma	1 (1%)
Signet-ring cell variants	2 (2%)
Anatomical site of uterine involvement, *n* (%) ^2^	
Endometrium	72 (69%)
Myometrium	27 (26%)
Cervix	28 (27%)
Endometrial polyp (substrate)	15 (14%)
Uterine leiomyoma (substrate)	8 (8%)
Concurrent ovarian involvement	8 (8%)
Fallopian tube	2 (2%)
Molecular profile	
ER status reported/ER-positive	60/54 (90%)
HER2 status reported/HER2-positive	38/15 (39%)
Triple-negative breast cancer	3 (3%)
Prior systemic and locoregional therapy, *n* (%) ^3^	
Tamoxifen	41 (39%)
Aromatase inhibitor	12 (11%)
Any endocrine therapy	58 (55%)
Cytotoxic chemotherapy	35 (33%)
Locoregional radiotherapy	18 (17%)
No prior therapy reported	12 (11%)
Presenting clinical features, *n* (%) ^4^	
Abnormal uterine/vaginal bleeding	62 (68%)
Asymptomatic at detection	18 (20%)
Pain, distension, or palpable mass	8 (9%)
Other	3 (3%)
Latency from primary to uterine metastasis	
Reported, *n* (%)	74 (70%)
Median (months)	36
Range (months)	−24–360
Synchronous (≤3 months), *n*	13
Occult primary, *n*	3
Very late (>10 years), *n*	8
Reported outcome ^5^	
Outcome reported, *n* (%)	29 (28%)
Died of disease	13 (45%)
Median survival in deaths (months)	6 (range 2–24)

^1^ Histology denominator (*n* = 88) excludes 17 cases reported as “breast carcinoma”, “breast adenocarcinoma”, or unspecified subtype. ^2^ Anatomical sites are not mutually exclusive; multifocal involvement was common. ^3^ Therapy categories overlap; multiple modalities per patient were possible. ^4^ Symptom denominator (*n* = 91) excludes 14 cases without explicit symptom reporting. ^5^ Outcome denominator (*n* = 29) excludes cases without follow-up reporting; survival in deaths is post-uterine-metastasis diagnosis.

**Table 2:** Comparison of anatomical and treatment variables between invasive lobular carcinoma (ILC) and invasive ductal carcinoma/invasive breast carcinoma not otherwise specified (IDC/IBC NOS) subgroups within the uterine metastasis cohort. Odds ratios with 95% CIs and *p*-values from Fisher’s exact test (two-sided); the Mann–Whitney U test was used for latency.

Variable	ILC (*n* = 51)	IDC/IBC NOS (*n* = 32)	OR (95% CI)	*p*-Value
Anatomical involvement				
Endometrium	38 (74.5%)	20 (62.5%)	1.75 (0.68–4.55)	0.33
Myometrium	12 (23.5%)	10 (31.2%)	0.68 (0.25–1.82)	0.46
Cervix	15 (29.4%)	9 (28.1%)	1.06 (0.40–2.83)	1.00
Polyp-substrate	11 (21.6%)	3 (9.4%)	2.66 (0.68–10.4)	0.23
Concurrent ovarian	5 (9.8%)	2 (6.2%)	1.63 (0.30–8.95)	0.70
Endocrine therapy exposure				
Tamoxifen	24 (47.1%)	13 (40.6%)	1.30 (0.53–3.18)	0.65
Aromatase inhibitor	8 (15.7%)	3 (9.4%)	1.80 (0.44–7.35)	0.52
Clinical context				
Asymptomatic detection	8 (15.7%)	8 (25.0%)	0.56 (0.19–1.68)	0.39
Median latency (months)	36	48	—	0.55 †

ILC = invasive lobular carcinoma; IDC = invasive ductal carcinoma; IBC NOS = invasive breast carcinoma not otherwise specified; OR = odds ratio; CI = confidence interval. † Mann–Whitney U test; latency available in 42 ILC and 27 IDC/IBC NOS cases. Negative latency values denote uterine metastasis identified before the breast primary. These subgroup comparisons are exploratory and were not powered to detect modest differences.

**Table 3:** Differential diagnosis of uterine lesions showing discohesive or atypical infiltrative growth in a breast cancer survivor. Morphological features alone are rarely diagnostic; the immunohistochemical profile is essential for confident distinction between metastatic breast carcinoma and the principal mimics. The panel listed for each entity represents the markers most useful in differential diagnosis rather than a comprehensive workup.

Entity	Morphological Features	Key Immunohistochemical Profile
Metastatic invasive lobular carcinoma	Discohesive single-file growth, small uniform cells with intracytoplasmic lumina, stromal infiltration with preserved native glandular architecture	GATA3+, TRPS1+, ER+, PR variable, E-cadherin loss, PAX8−, CK7+, CK20−, CDX2−
Metastatic invasive breast carcinoma of no special type	Diffuse or nodular growth of polygonal cells, variable nuclear pleomorphism, may mimic poorly differentiated primary uterine carcinoma	GATA3+, TRPS1+, ER variable, E-cadherin retained, PAX8−, CK7+, CK20−, CDX2−
Primary endometrioid endometrial carcinoma	Confluent glandular and cribriform architecture, columnar cells with apical mucin, may show squamous differentiation	PAX8+, ER+, PR+, GATA3−, TRPS1−, E-cadherin retained, vimentin+
Primary uterine serous carcinoma	Papillary or solid architecture, high-grade pleomorphic nuclei, frequent mitoses and apoptosis	PAX8+, p53 aberrant (overexpression or null), WT1 variable, GATA3−, TRPS1−, ER often weak/negative
Endometrial stromal sarcoma	Monomorphic small cells resembling proliferative endometrial stroma, characteristic spiral arteriole-like vessels, tongue-like myometrial invasion	CD10+, ER+, PR+, cyclin D1 variable, GATA3−, TRPS1−, cytokeratin negative or focal
Lymphoma involving the uterus	Diffuse discohesive infiltrate of monomorphic cells, may mimic stromal sarcoma or metastatic lobular carcinoma at low power	CD45 (LCA)+, lineage markers (CD20, CD3) positive depending on type, all epithelial markers (cytokeratin, GATA3, TRPS1) negative
Metastatic gastrointestinal signet-ring carcinoma	Discohesive single cells or small clusters with prominent intracytoplasmic mucin, may closely mimic metastatic lobular carcinoma	CK20+, CDX2+, CK7 variable, GATA3−, TRPS1−, ER−, mucicarmine and PAS-diastase positive
Metastatic urothelial carcinoma	Nests and clusters of pleomorphic cells, occasional squamoid features, may be uropathic in setting of bladder or upper tract primary	GATA3+, p63+, CK7+/CK20 variable, uroplakin II+, TRPS1− (typically), ER−, PAX8−

GATA3 = GATA-binding protein 3; TRPS1 = trichorhinophalangeal syndrome 1; ER = estrogen receptor; PR = progesterone receptor; PAX8 = paired-box gene 8; CK7 = cytokeratin 7; CK20 = cytokeratin 20; CDX2 = caudal-type homeobox 2; CD10/CD20/CD3/CD45 = cluster of differentiation antigens; LCA = leucocyte common antigen; WT1 = Wilms tumour 1; PAS = periodic acid–Schiff. The breast lineage panel (GATA3, TRPS1) is the most discriminating component in most differentials; PAX8 reliably excludes breast origin when positive. TRPS1, although a sensitive breast marker, is not entirely breast-specific—nuclear expression can occur in a subset of gynaecological and other tumours—and should therefore be interpreted as part of the panel rather than in isolation.

**Table 4:** Key clinical and diagnostic takeaways from this quantitative review of uterine metastasis from breast carcinoma.

Aspect	Key Takeaway
Commonest histology	Invasive lobular carcinoma (~58% of cases), an approximately 4–6-fold over-representation relative to its frequency among primary breast cancers.
Typical presentation	Abnormal uterine bleeding in ~68% of cases; around 20% are asymptomatic and detected incidentally on surveillance imaging or in a polyp or curettage specimen.
Commonest site	Endometrium (~69%); the cervix and myometrium are each involved in ~26–27%; multifocal uterine and adnexal involvement is frequent.
Key risk context	Tamoxifen-associated endometrial polyps are strongly enriched for metastatic deposits (OR ~8.4); every polyp removed in an exposed survivor warrants careful histological and immunohistochemical scrutiny.
Most informative immunohistochemistry	GATA3 and/or TRPS1 positivity with PAX8 negativity favours breast origin; loss of E-cadherin supports a lobular phenotype; direct comparison with the original breast tumour remains the most reliable confirmation.
Main diagnostic pitfalls	Deposits may mimic endometrial stromal sarcoma, poorly differentiated or serous carcinoma, and lymphoma; TRPS1 and mammaglobin are not wholly specific for breast origin.
Latency and surveillance	The median interval from breast cancer to uterine metastasis is ~3 years but extends up to ~30 years, particularly for lobular carcinoma, justifying a low threshold for sampling uterine lesions in any breast cancer survivor.

## Data Availability

The complete case-level dataset underlying this review (105 cases extracted from the published literature) is provided as Supplementary Table S1. Additional details from the original published case reports cited herein are available from the corresponding author upon reasonable request.

## Supplementary Materials

The following supporting information can be downloaded at: https://www.mdpi.com/article/10.3390/medicina62061205/s1. Figure S1, Literature search and case selection flow diagram; Figure S2, Forest plot of odds ratios for the tamoxifen–polyp association across primary and sensitivity analyses; Table S1, Case-level dataset of the 105 published cases of uterine metastasis from breast carcinoma (1982–2025); Table S2, Corpus-level methodological appraisal of the included case-report literature, performed using the framework of Murad et al. [20].

## Abbreviations

The following abbreviations are used in this manuscript: AE1/AE3, pancytokeratin (AE1 + AE3 antibody cocktail); CDH1, cadherin-1 gene; CDK4/6, cyclin-dependent kinase 4/6; CDX2, caudal-type homeobox 2; CI, confidence interval; CK7, cytokeratin 7; CK20, cytokeratin 20; DOD, died of disease; ER, estrogen receptor; GATA3, GATA-binding protein 3; GCDFP-15, gross cystic disease fluid protein 15; HER2, human epidermal growth factor receptor 2; IBC NOS, invasive breast carcinoma not otherwise specified; IDC, invasive ductal carcinoma; IHC, immunohistochemistry; ILC, invasive lobular carcinoma; MMR, mismatch repair (proteins); OR, odds ratio; PAX8, paired-box gene 8; PET-CT, positron emission tomography–computed tomography; PR, progesterone receptor; T-DM1, trastuzumab emtansine; TRPS1, trichorhinophalangeal syndrome 1.
