# Supplementary material for: When Breast Cancer Meets the Uterus: A Quantitative Review of 105 Cases Spanning Four Decades
Source: Medicina (Kaunas). 2026 Jun 22;62(6):1205. doi: 10.3390/medicina62061205 (PMC13304205; doi:10.3390/medicina62061205)
Supplement: Supplementary file 1 [file medicina-62-01205-s001.zip › table_s2.pdf]

Supplementary Table S2. Corpus-level methodological appraisal of the included case-report literature

Manuscript medicina-4344453 — "When Breast Cancer Meets the Uterus: A Quantitative Review of 105 Cases Spanning Four Decades." The body of evidence (n = 105 published cases) was appraised collectively against the framework of Murad and colleagues for the methodological quality of case reports and case series, across four domains — selection, ascertainment, causality, and reporting. Because this framework is designed for non-comparative reports, it is applied here at the level of the corpus rather than scored per individual case.

Domain	Appraisal question (Murad framework)	Corpus-level assessment	Basis in the present case series (n = 105)
Selection	1. Do the reported patients represent the whole, unselected experience of the investigators, or is the selection process unclear such that similar cases may have gone unreported?	**High risk of selection bias**	The corpus consists almost entirely of single-patient case reports and small case series. Individual reports carry no defined denominator and do not reflect the unselected experience of any centre; cases are preferentially published when unusual, complete, or instructive, and asymptomatic or uncomplicated cases are likely under-reported.
Ascertainment	2. Was the exposure adequately ascertained?	**Adequate for the primary diagnosis; partial for treatment exposure**	A histologically confirmed breast carcinoma primary was a prerequisite for inclusion and was documented in all 105 cases. The histological subtype of the primary was specified in 88 of 105 cases (84%), the molecular/receptor profile in 61 of 105 (58%), and prior systemic or locoregional therapy in 83 of 105 (79%). Tamoxifen exposure specifically was unreported in a subset of cases, addressed by the sensitivity analyses in the main text.
	3. Was the outcome adequately ascertained?	**Adequate for the index event; inadequate for clinical outcome**	The uterine metastatic deposit — the index event of this review — was confirmed histologically (with supporting immunohistochemistry in the majority) in all included cases. By contrast, clinical follow-up or survival outcome of any kind was reported in only 29 of 105 cases (28%).
Causality	4. Were alternative explanations for the observation (e.g., a primary uterine or ovarian malignancy) excluded?	**Adequate in well-characterised cases; limited in older reports**	Distinction of metastatic breast carcinoma from a primary uterine or ovarian tumour was supported by immunohistochemistry (GATA3 and/or TRPS1 positivity with PAX8 negativity, alongside correlation with the known breast primary) in most contemporary reports. Older reports frequently predated this panel, so an alternative primary could not always be formally excluded.
	5. Was a challenge / rechallenge phenomenon present?	**Not applicable**	Uterine metastasis is an observed disease event rather than an exposure that can be withdrawn and re-administered; a challenge/rechallenge paradigm does not apply to this question.
	6. Was a dose–response relationship demonstrable?	**Not applicable**	The data do not permit assessment of a dose–response relationship between any exposure (including tamoxifen dose or duration) and the occurrence of uterine metastasis; dose and duration were inconsistently reported.
	7. Was follow-up long enough for the outcome of interest to manifest?	**Adequate for the index event; insufficient for survival outcomes**	Follow-up was sufficient to ascertain the index event (the metastasis) in all cases. Follow-up for survival was frequently short or absent, and long-surviving patients are systematically more likely to be reported, so survival estimates derived from the reported subset are subject to reporting bias and are not presented as prognostic.

**Reporting**	8. Are the cases described in sufficient detail to allow replication or practical inference?	**Adequate in recent reports; sparse in older reports**	Recent case reports generally provide sufficient histological, immunohistochemical, treatment, and follow-up detail to permit replication and practical inference. Older reports are frequently sparse, lacking molecular profile, latency, or outcome data; this missingness is non-random and concentrated in the earlier literature.

Overall assessment. The corpus carries a high risk of selection and reporting bias inherent to case-report literature. Ascertainment of the histological diagnosis is adequate — both of the breast primary and of the uterine deposit — but ascertainment of treatment exposure, molecular profile, and clinical outcome is limited and concentrated in the more recent literature. The quantitative estimates in this review should therefore be interpreted as descriptions of the published case literature rather than as population-level parameters.

Appraisal framework. Murad MH, Sultan S, Haffar S, Bazerbachi F. Methodological quality and synthesis of case series and case reports. BMJ Evidence-Based Medicine. 2018;23(2):60–63. doi:10.1136/bmjebm-2017-110853.

Note. *'Not applicable' denotes a framework item that does not pertain to the observation of a metastatic event (as opposed to an item that was assessable but unmet). Completeness percentages are computed over the full set of 105 included cases.*
