# Supplementary figures and images for: When Breast Cancer Meets the Uterus: A Quantitative Review of 105 Cases Spanning Four Decades
Source: Medicina (Kaunas). 2026 Jun 22;62(6):1205. doi: 10.3390/medicina62061205 (PMC13304205; doi:10.3390/medicina62061205)

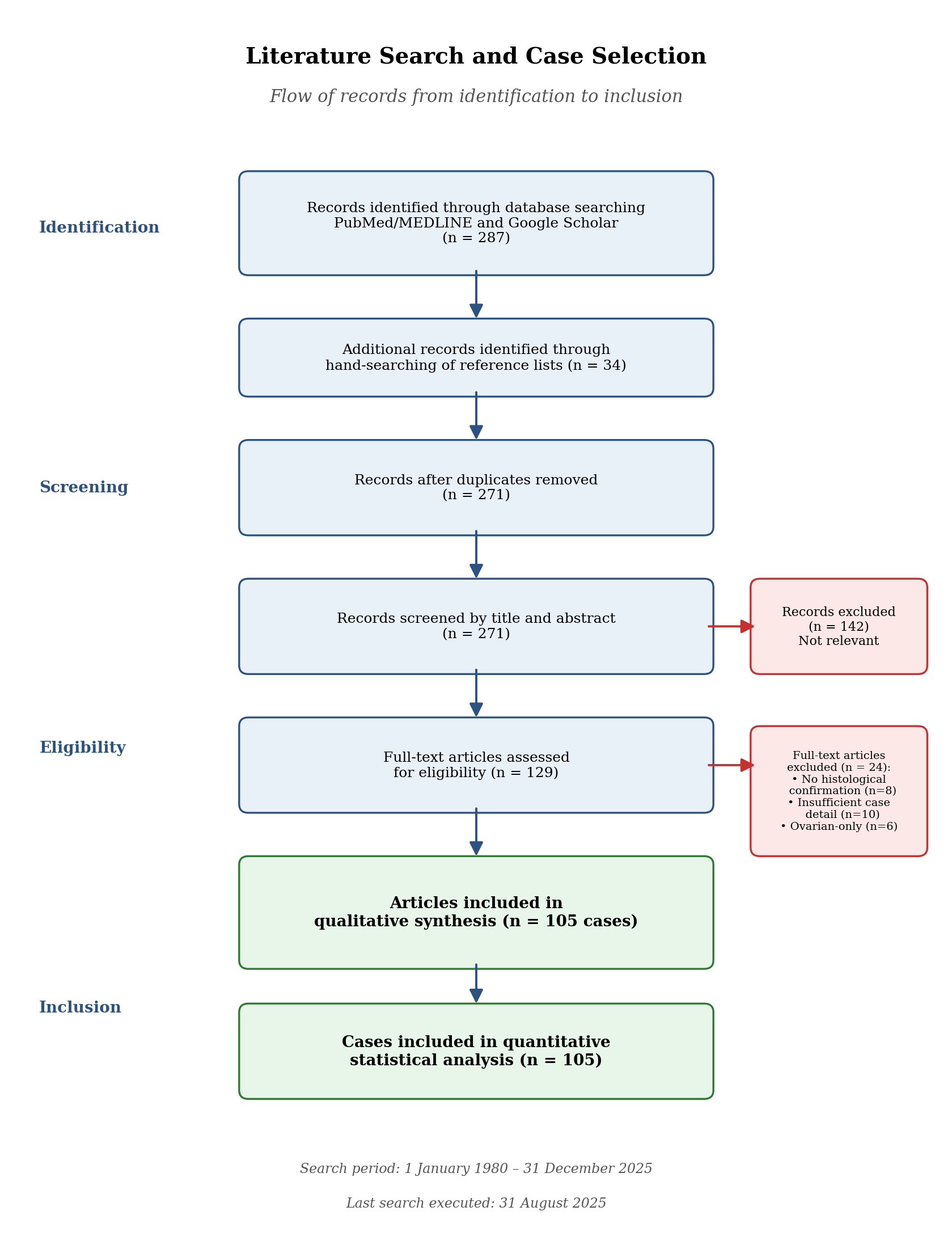

Supplement: Supplementary file 1 [file medicina-62-01205-s001.zip › figure_s1_search_flow.png]

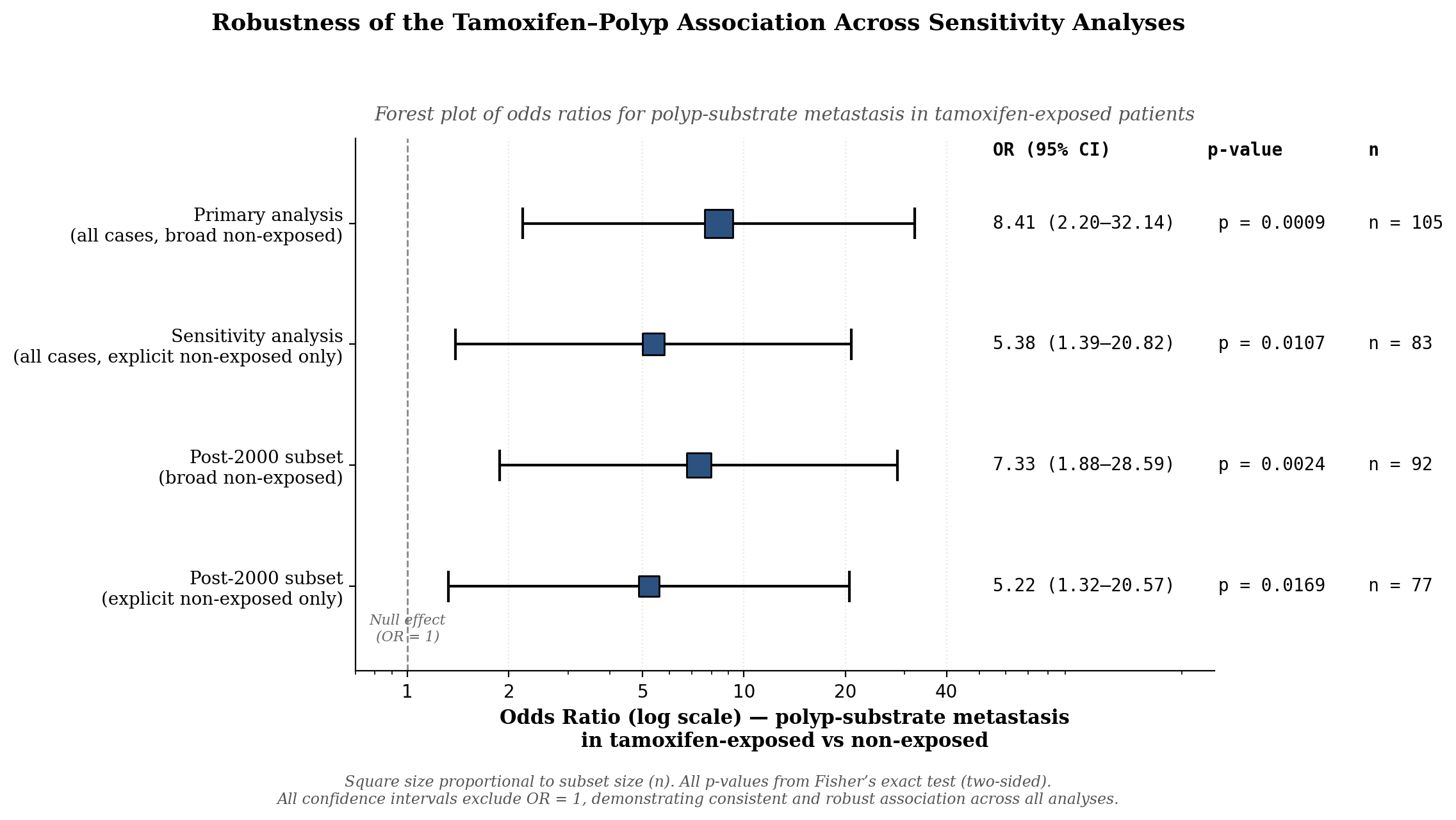

Supplement: Supplementary file 1 [file medicina-62-01205-s001.zip › figure_s2_forest_plot.png]
